# Economic evaluation of rivaroxaban in stroke prevention for patients with atrial fibrillation in Greece

**DOI:** 10.1186/1478-7547-12-5

**Published:** 2014-02-10

**Authors:** Georgia Kourlaba, Nikos Maniadakis, George Andrikopoulos, Panos Vardas

**Affiliations:** 1 The Stavros Niarchos Foundation-Collaborative Center for Clinical Epidemiology and Outcomes Research (CLEO), Thivon & Papadiamantopoulou, Goudi, Athens 115 27, Greece; 2 Department of Health Services Organisation & Management, National School of Public Health, Athens, Greece; 3 Cardiology Department, Henry Dunant Hospital, Athens, Greece; 4 Department of Cardiology, Heraklion University Hospital, University of Crete, Heraklion, Greece

**Keywords:** Cost-effectiveness, Vitamin-K-antagonists, Cost-utility

## Abstract

**Background:**

To undertake an economic evaluation of rivaroxaban relative to the standard of care for stroke prevention in patients with non-valvular atrial fibrillation (AF) in Greece.

**Methods:**

An existing Markov model designed to reflect the natural progression of AF patients through different health states, in the course of three month cycles, was adapted to the Greek setting. The analysis was undertaken from a payer perspective. Baseline event rates and efficacy data were obtained from the ROCKET-AF trial for rivaroxaban and vitamin-K-antagonists (VKAs). Utility values for events were based on literature. A treatment-related disutility of 0.05 was applied to the VKA arm. Costs assigned to each health state reflect the year 2013. An incremental cost effectiveness ratio (ICER) was calculated where the outcome was quality-adjusted-life year (QALY) and life-years gained. Probabilistic analysis was undertaken to deal with uncertainty. The horizon of analysis was over patient life time and both cost and outcomes were discounted at 3.5%.

**Results:**

Based on safety-on-treatment data, rivaroxaban was associated with a 0.22 increment in QALYs compared to VKA. The average total lifetime cost of rivaroxaban-treated patients was €239 lower compared to VKA. Rivaroxaban was associated with additional drug acquisition cost (€4,033) and reduced monitoring cost (-€3,929). Therefore, rivaroxaban was a dominant alternative over VKA. Probabilistic analysis revealed that there is a 100% probability of rivaroxaban being cost-effective versus VKA at a willingness to pay threshold of €30,000/QALY gained.

**Conclusion:**

Rivaroxaban may represent for payers a dominant option for the prevention of thromboembolic events in moderate to high risk AF patients in Greece.

## Background

Atrial Fibrillation (AF) constitutes a significant public health problem as it is strongly associated with increased risk of morbidity, such as stroke, heart failure, left ventricular dysfunction and thromboembolism, increased risk of mortality and reduced quality of life (QoL) [1-3]. Stroke is the most devastating and feared complication of AF, and in the absence of anti-thrombotic therapy the annual risk of stroke in patients with non-valvular AF increases from about 5% in patients less than 65 years of age to about 8% in patients 75 years of age or older [1]. Along with mortality and morbidity, AF imposes a great economic burden which stems from the excess health care resources utilization related to the management of the disease as well as its complications [4-7].

Taking into account the aforementioned, the need for effective and efficient management of AF patients is evident. The care of AF patients should be focused on reducing the symptoms and preventing severe AF-related complications. The guidelines issued by the European Society of Cardiology and American Heart Association as well as local guidelines issued recently by the National Medicines Agency, advocate that antithrombotic therapy, including oral anti-coagulants (OAC), anti-platelets and combination of OAC with anti-platelets, represent good options in the effective prevention of complications associated with AF [8-10]. Moreover, guidelines suggest that the management of AF patients should vary according to their risk status for stroke, defined by the CHADS2 (Cardiac failure, Hypertension, Age, Diabetes, Stroke2) Index [11]. Specifically, patients with medium to high risk of stroke should be receiving OAC, such as vitamin K antagonists (VKA) including warfarin and acenocoumarol. A recent meta-analysis revealed that VKA-treated patients experience significantly lower risk for stoke compared to those receiving placebo or remain untreated [12]. However, VKA anti-coagulation has a narrow therapeutic window and requires regular blood monitoring to ensure that patients are within optimal therapeutic ranges, defined as International Normalised Ratios (INRs) between 2.0 and 3.0. In addition, OACs have a number of known drug and food interactions, and thus maintaining patients in the narrow therapeutic window of INR 2-3 is difficult. Therefore, there is a need for effective and safe OACs that are simpler to administer.

Rivaroxaban (Xarelto®), an oral direct factor Xa inhibitor, represents a new OAC option for use in this group of patients [13]. The “Rivaroxaban Once Daily Direct Factor Xa Inhibition Compared with Vitamin K Antagonism for Prevention of Stroke and Embolism Trial in Atrial Fibrillation” (ROCKET AF) was designed to determine whether rivaroxaban (at a daily dose of 20 mg) is non-inferior to dose-adjusted VKA for the primary end point of stroke (ischaemic and haemorrhagic stroke) or systemic embolism (SE) [14,15]. In the safety on-treatment analysis (SOT), rivaroxaban demonstrated a 21% relative risk reduction for stroke and SE compared with warfarin (hazard ratio 0.79, 95% confidence interval [CI] 0.65–0.95, p = 0.02 for superiority test) [15]. Thus, it represents an effective alternative for stroke prevention, but it also may impose a considerable cost to the health care system and payers.

The recent climate of the major financial crisis has resulted in strong health care budgetary constraints and more than ever before emerges the need to increase the efficiency in health care delivery and the cost-effectiveness of interventions. In this light, the aim of the present study was to conduct an economic evaluation comparing rivaroxaban (Xarelto®) with the standard therapy (i.e. VKAs) for stroke prevention in patients with AF in the Greek healthcare setting.

## Methods

In the present study, an existing Markov model evaluating the lifetime cost-effectiveness of rivaroxaban relative to selected antithrombotic alternatives for stroke prevention in AF patients was adapted to the Greek health care setting. The analysis was conducted from the Greek third-party payer perspective. Costs and outcomes that occur beyond one year were discounted at a 3.5% annual rate which is the standard practice in these studies in Greece [16].

### Model structure

**Figure 1:**
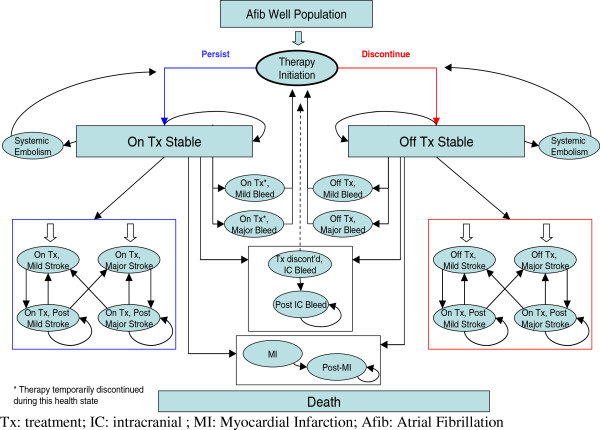
Model structure.

The model was designed to reflect the natural progression of AF patients through different health states, during the course of three month cycles and its aim was to sufficiently capture the frequency of major events, up to death. The model structure is depicted in simplified form in Figure 1. In particular, patients enter the model with stable uncomplicated AF and are being treated with rivaroxaban or VKAs. Then, in each subsequent three month cycle during their life span, patients can either remain without any event or experience one of the following complications: major and minor ischemic stroke (IS), systemic embolism (SE), myocardial infarction (MI), major and minor extracranial (EC) bleeding, intracranial (IC) bleeding or death from AF-related cause or other causes. The major events may be classified as transient events or events with permanent after-effects. The IC bleed event may result in discontinuation of the current OAC treatment depending on the stroke risk of the population. Patients cannot experience two acute complications in the same cycle. Patients can also switch or discontinue treatment permanently for reasons not related to a clinical event.

### Patient population

The hypothetical cohort of 1000 patients entering the Markov model is assumed to be representative of the Greek patients with non-valvular AF and with at least one risk factor for stroke. Based on data obtained from an ongoing study entitled “Current Clinical Practice in the Management of Atrial Fibrillation in Greece: The MANAGE-AF study” the mean age of AF patients in Greece is 75 years (“data on file”). Therefore, 75 years was used as the starting age in the model. At this point, it should be mentioned that the UK’s National Institute of Health and Clinical Excellence (NICE) concluded that there is no reason to believe that the efficacy/safety extracted from ROCKET-AF would be different in patients at low risk of stroke based on the pharmacokinetics [17]. Sub-populations such as those at high risk of stroke, those with prior stroke, poorly controlled VKA-patients as well as VKA-naïve patients were considered in sensitivity analyses.

### Comparators

The most widely used antithrombotic alternatives in the common clinical practice for stroke prevention in AF patients were considered as comparators of rivaroxaban in the present analysis. In particular, adjusted-dose VKA (i.e. acenocoumarol, up to 2.5 mg per day, that is widely used in local clinical practice instead of warfarin), with a target therapeutic INR of 2.0 to 3.0, represents the standard of care. In the case of a discontinuation, it is assumed that patients who received either rivaroxaban or VKA are switched to aspirin monotherapy. An alternative scenario where both rivaroxaban and VKA-treated patients are switched to no treatment was also considered in sensitivity analysis.

### Model inputs

In each different health state a treatment cost is attached, in order to estimate the total and the mean cost of therapy, based on the progression of patients from the original cohort through different health states. In a similar manner, each health state is also associated with a utility weight, which reflects quality of life in that particular state, and is used in order to estimate total and mean lived years with and without quality of life adjustment.

#### Efficacy and safety data

In a Markov model, patients move from one health state to another with a certain probability called “transition probability”. Transition probabilities for all stages of a treatment cycle are usually obtained from available clinical trials, observational studies and meta-analyses. In the current study, transition probabilities were calculated based on event rates and relative treatment effects (RRs) obtained from the phase III ROCKET AF trial [15]. The quarterly VKA rates for IS, SE, bleeding events and MI obtained from the SOT analysis of the ROCKET AF were used as baseline rates [15]. In particular, the quarterly rates were calculated based on the corresponding annual rates reported in the ROCKET AF trial by the following formula: Quarterly rate = 1 − (1 − annual rate) ^ ^1/4^ (Table 1).

**Table 1:** Event rates and relative risks of clinical events for each therapy

	**3-month baseline rate**	**Sources**
Ischemic stroke		
VKA	0.40%	ROCKET AF [15]^§^
Myocardial infarction		
VKA	0.28%	ROCKET AF [15]^§^
Systemic embolism		
VKA	0.05%	ROCKET AF [15]^§^
Intracranial bleeding		
VKA	0.19%	ROCKET AF [15]^§^
Minor extracranial bleeding		
VKA	2.97%	ROCKET AF [15]^§^
Major extracranial bleeding		
VKA	0.69%	ROCKET AF [15]^§^
	**RR (95% CI)**	**Sources**
Ischemic stroke		
Rivaroxaban vs. VKA (SOT)	0.94 (0.75 – 1.17)	ROCKET AF [18]
Rivaroxaban vs. VKA (ITT)	0.99 (0.82 – 1.20)	ROCKET AF [15]
Myocardial infarction		
Rivaroxaban vs. VKA (SOT)	0.81 (0.63 – 1.06)	ROCKET AF [15]
Rivaroxaban vs. VKA (ITT)	0.91 (0.72 – 1.16)	ROCKET AF [15]
Systemic embolism		
Rivaroxaban vs. VKA (SOT)	0.23 (0.09 – 0.61)	ROCKET AF [18]
Rivaroxaban vs. VKA (ITT)	0.74 (0.42 – 1.32)	ROCKET AF [15]
Intracranial bleeding		
Rivaroxaban vs. VKA	0.67 (0.47 – 0.93)	ROCKET AF [15]^§^
Major extracranial bleeding		
Rivaroxaban vs. VKA	1.14 (0.98 – 1.33)	ROCKET AF [18]^§^
Minor extracranial bleeding		
Rivaroxaban vs. VKA	1.04 (0.96 – 1.13)	ROCKET AF [15]^§^
	**Mortality rates**^**¶**^	**Sources**
**Stroke related**		
Case fatality of major stroke	12.6%	ROCKET AF [15]
Case fatality of minor stroke	0.00%	Assumption
Post-major stroke mortality rate	2.60%	Marini et al. 2005 [19]
Post-minor mortality rate	0.00%	Assumption
**Bleeding-related**		
Case-fatality of major bleed	1.60%	ROCKET AF [15]
Case-fatality of minor bleed	0.00%	Assumption
Case-fatality of intracranial bleed	38.80%	ROCKET AF [15]
Post- major bleed mortality rate	0.00%	Assumption
Post- minor bleed mortality rate	0.00%	Assumption
Post- Intracranial bleed mortality rate	2.60%	Assumption (equal to stroke)
**Systemic embolism-related**		
Case-fatality of systemic embolism	0.00%	Eckman 1992 [20]
**MI-related**		
Case-fatality rate of myocardial infarction	9.69%	ROCKET AF [15]
Post myocardial infarction mortality rate	2.68%	Hoit et al. 1986 [21]

*SOT* safety on treatment, *ITT* Intention-to-Treat, *VKA* vitamin-K-antagonist.

^**¶**^based on the sum of case fatality rates in both arms of the ROCKET trial.

^§^Data derived from safety on treatment analysis of ROCKET trial.

Relative treatment effects (RRs) describing the efficacy of rivaroxaban versus VKA for IS, SE, bleeding events and MI were extracted from the ROCKET AF [15,18]. In the base case analysis, the RRs for IS, MI, SE and bleed derived from SOT analysis of the ROCKET AF trial data were considered, to be comparable with previous relative studies [22,23], while the RRs for IS, MI and SE obtained from intention-to-treat (ITT) analysis were used in sensitivity analysis. Because bleed events are safety endpoints, it was not appropriate to also consider RRs from the ITT population (Table 1). At this point, it should be noted that for the current analysis where the discontinuation is being modelled (i.e. event rates on and off-treatment), the most appropriate data to use depend on the treatment assumptions to avoid the double-counting of the impact of discontinuation on events. More specifically, as long as patients are on treatment the SOT data are most relevant, once off-treatment data related to the selected second line treatment must be applied (e.g. aspirin from an additional external source).

After major IS, major EC bleeding events, MI and IC bleeding events, patients may die with a probability equal to the case-fatality rate extracted from the SOT analysis of ROCKET AF trial data [15]. This case fatality rate was based on the average of case fatality rates in both arms of the ROCKET AF trial, thus making the assumption that the case fatality is the same, no matter which treatment the patient is receiving [15]. Minor IS and minor bleeding events with no lasting sequelae as well as the SE were assumed to have no associated case-fatality. For patients who have experienced a major IS and a MI, there is evidence for an impact on life expectancy, which means an increased mortality rate even after recovery from the acute episode. Since post-recovery mortality data is not available for the ROCKET AF trial, these mortality rates were estimated based on literature [19,21]. Moreover, since no literature on post-IC bleed mortality rates was found, the increase in subsequent mortality rates was assumed to be equal to that following a major IS [19]. Finally, patients recovering from a major bleed are expected to fully recover over the duration of three months, and thus no increase in subsequent mortality rate has been assumed (Table 1).

### Discontinuation and switching

Persistence indicates whether or not patients continue or discontinue their current treatment. As an initial step towards adjusting the efficacy which is associated with a clinical trial, permanent discontinuation rates in an observational setting can be used to describe the proportion of patients who cease to receive treatment entirely.

Although limited data for the real-life discontinuation rate is available for rivaroxaban, the model currently uses discontinuation rates from the ROCKET AF trial [15] as the real-life available data indicates that the discontinuation rate for the first 3-month follow up period is extremely close to that obtained from ROCKET data (9.1% and 8.9%, respectively). In this context, the discontinuation rate was set at 8.9% and 8.0% for rivaroxaban and VKA, respectively as concerns the initial cycle and at 4.39% and 4.46%, respectively in subsequent cycles, based on data from the ROCKET AF trial. Discontinuation probabilities were calculated based on the proportion of patients persisting on therapy at 3 months (x) and 12 months (y), using the following formulae:

Discontinuationat3months=1−x

Subsequentdiscontinuation=1−1−y−x^1/3.

#### Utility values

Utility values describe the health-related QoL associated with different health states on a scale of zero to one, where zero is equivalent to death and one represents best imaginable health. Due to lack of local utility values for patients with AF on or off anti-thrombotic therapy, the values proposed by published studies in the literature were considered in the present analysis [24-30]. These values have been obtained by measuring QoL of these patients with the EQ-5D instrument. In particular, a utility value of 0.779 was assigned to the baseline untreated state of an AF patient [31]. The same value was assigned for those receiving rivaroxaban as it was assumed that there is no disutility associated with this therapy. On the other hand, a treatment-related disutility of 0.05 was applied to VKA. This was obtained from a study evaluating how patients with AF (attending GP- and hospital-led clinics) value different health outcomes [25]. An alternative scenario with no VKA-related disutility in the model was considered in the sensitivity analysis. The utility values of 0.189, 0.641 and 0.680 were assigned to the three health states describing the acute episodes of major and minor IS as well as MI [25,32], while the values of 0.72, 0.48, and 0.69 were considered for the post minor and post major IS and post MI [26,33]. As concerns bleeding events, the utility values of 0.776 and 0.598 were assigned to minor and major bleeds [24], while a value of 0.60 was used for IC bleed events and 0.74 to reflect QoL of patients in the post IC bleed event period [27,28]. Finally, a value of 0.66 was used in association to the systemic SE state [24].

### Estimation of costs

The perspective of the economic evaluation was that of the public third-party-payer and as such only health care costs reimbursed by the payer were considered in the analysis and any other cost, such as the cost related to the central Government budget to cover personnel salaries or patient co-payments, were not considered. In particular, drug acquisition costs, monitoring costs, event treatment-related costs (acute and follow-up costs), patient reimbursed transportation costs were the main ones considered in the model. All costs reflect the year 2013.

The reimbursement costs to payers associated with acute treatment of IS, SE, MI, IC and EC bleeding events were obtained from the corresponding Diagnostic Related Group (DRG) tariffs, which were issued recently by the Greek Ministry of Health [34]. A major IS, IC bleeding and MI also incurred a cost associated with follow-on care for the rest of patients’ life. The 3-month cost of care for IS and MI was extracted from a published study which detailed different components of the follow-up cost of patients with stroke and MI in Greece (Table 2) [35]. The follow-up cost for IC bleeding was assumed to be identical to corresponding cost for a major IS. With regards to major EC bleedings and SE, it was assumed that there were no subsequent costs after 3 months.

**Table 2:** Cost inputs used in the base case analysis

**Cost inputs**	**Costs (€)**	**Sources**
**Drug acquisition cost (per day)**		
Rivaroxaban	2.16	Drug price bulletin [36]
VKA (Acenocoumarol)	0.05	Positive Drug List [37]
**Monitoring cost**		
VKA monitoring visit (1^st^ visit)	32	Government Gazzette
VKA monitoring visit (subsequent visits)	22	Government Gazzette
Other therapies monitoring visit	10	Government Gazzette
**Acute treatment event cost**		
Minor ischemic stroke	900	DRGs (N30X)
Moderate ischemic stroke	1625	DRGs (N30Mβ)
Severe ischemic stroke	2475	DRGs (N30Mα)
Systemic embolism	1567	DRGs (K45Mβ, K45Mα)^§^
Intracranial haemorrhage	2,475	DRGs (N30Mα)
Minor extracranial haemorrhage	257	DRGs (Ξ21X)
Major extracranial haemorrhage	654	DRGs (Π41M, Π41X)^§^
Acute myocardial infarction	1,783	DRGs (K10M, K10X, K31M, K31X, K40M, K40X)^§^
**Follow up cost per three month cycle**		
Major stroke	1,093	THESIS study [35]
Intracranial haemorrhage	1,093	Assumption
Myocardial infarction	1,296	THESIS study [35]

All costs reflect the year 2013.

^§^weighted mean of related DRGs has been calculated taking into account the proportion of patients belongs to each DRG code, based on expert’s opinion.

*VKA* vitamin-K-antagonist, *DRG* Diagnostic Related Group.

The cost of medications was estimated on the basis of the defined daily dose and corresponding cost. In the base case analysis, the drug daily cost was calculated on grounds of the social security reimbursement price, defined by the internal reference price system attached to the latest published positive drug list (May 2013) [37]. In particular, when the drug retail price was higher than the corresponding reimbursement one, the payer cost was calculated based on the reference price minus the patient co-payment (25%), plus 50% of the difference between reference price and retail prices. On the other hand, when the retail price was lower than the reference price, the payer cost was obtained from the reference price minus the patient co-payment. In the absence of social security reimbursement price (i.e. in case of rivaroxaban), the available retail price published in the latest drug price bulletin issued by the Greek Ministry of Health was used (August 2013) [36]. Moreover, there is a rebate of 9% imposed upon manufacturers to get into the positive drug list and then there is one more based on volume which can be up to 8% depending on quarterly sales (law 4052/2012, Government Gazzette). At this point, it should be noted that a conservative 5% volume-related rebate was considered on top of the 9% rebate to enter the positive list.

The monitoring cost is associated with physician monitoring visits and utilization of INR tests to evaluate the effectiveness of therapy. Rivaroxaban is fixed-dose oral therapy that does not require any blood monitoring. Therefore, the monitoring cost related to this therapy is equal only to the visit cost, which is assumed to be two per year, based on expert opinion. On the other hand, VKAs require regular INR monitoring and dose-titration over the duration of therapy. Especially, when patients are initiated on VKA for the first time, or after a period of therapy interruption, it is recommended that they see a physician regularly and frequently in order to adjust the dose of VKA until they have achieved stabilisation in their INR. Based on local expert opinion, one visit per week (including an INR test) is required during the first month and then once monthly. The per visit cost of a monitoring visit in Greece as well as the cost of INR test were obtained from the Government Gazzette (Table 2).

In addition, the cost of using patient transport services to attend the VKA monitoring clinics was incorporated. Some proportion of patients makes use of the third-party payer-sponsored patient transport service (PTS) for their transportation. A conservative 50% of patients were assumed to use this service, and a mean transportation cost of €70 per visit was considered based on a previous analysis regarding average distance and travelling cost per patient and the cost to the sickness funds [38]. A zero transportation cost was also considered in the sensitivity analysis.

### Data analysis

The aforementioned approach and data were used to get mean estimates of life time costs, LYs and QALYs for each comparator. When new options like rivaroxaban are more effective (i.e. higher QALY) and less costly than comparators, they are considered as “dominant” treatments. In cases where new options are less effective and more costly they are considered as “dominated” by the alternatives. In cases where they are associated with higher QALY and higher cost they are considered as cost-effective only when the ICER is lower than a specific predetermined willingness-to-pay (WTP) threshold (i.e. €40,000/QALY which is a commonly used standard or €60,000/QALY that corresponds to three times per capita GDP in Greece, as recommended by the World Health Organisation) [39-41]. There is no official WTP threshold for Greece and in published studies the above thresholds are often quoted. However, in the context of the economic crisis a much lower WTP threshold (i.e. €30,000/QALY) was considered.

The majority of input data used in the current model are subjected to variation. Therefore, a probabilistic sensitivity analysis (PSA) was conducted to test second-order uncertainty in the model. Hence, baseline risks were assumed to have beta distributions, while relative risks were assumed to be log-normally distributed. Event costs and utilities were assumed to have gamma and beta distributions, respectively. Simulation modeling was used to run 1000 iterations of the model each time varying all model parameters. The results of PSA are presented as cost-effectiveness acceptability curves (CEAC), which indicate the likelihood of the incremental cost per QALY to fall below specified WTP thresholds.

One way sensitivity analysis was also undertaken to test the robustness of the results, by varying individual parameters between low and high values, within plausible ranges, in order to ascertain the key drivers of cost-effectiveness. For parameters such as those reflecting clinical efficacy, where values were based on robust studies or reviews, the reported 95% CI were used. For parameters for which there are uncertainties surrounding the source data, ranges reported in the literature were used. Due to the large number of input parameters included in the model, only parameters that altered the ICER by more than €1000 were reported. All statistical calculations were performed using Microsoft Excel 2003.

## Results

### Base case analyses

The results of the base case cost-effectiveness analysis are presented in Table 3. Based on SOT data from the ROCKET AF trial, the analysis predicted a discounted QALY of 6.50 and 6.28 in rivaroxaban- and VKA-treated patients. The total lifetime cost was lower in the rivaroxaban arm (€7,868) compared to the VKA arm (€8,107), resulting in a cost saving of €239. Rivaroxaban was associated with additional drug acquisition cost (€4,033), however this was totally offset by reduced monitoring (-€3,929) and event management (-€341) costs. Therefore, rivaroxaban seems to be a dominant alternative over VKA, as the former is related with lower cost and greater health benefit.

**Table 3:** Cost-effectiveness results of base case analysis (SOT analysis of ROCKET AF trial data)

	**Rivaroxaban**	**VKA**
**Costs**		
Total costs (€)	7,868	8,107
Drug acquisition cost (€)	4,156	126
Drug monitoring costs (€)	52	3,981
Event treatment costs (€)	3,659	4,000
**Health outcomes**		
QALYs	6.50	6.28
LYs	8.55	8.48
Strokes	0.22	0.23
Bleeds	1.10	1.06
**Incremental analysis**		
ICER per QALY gained (€)	NA	Riv. Dom
ICER per LY gained (€)	NA	Riv. Dom

It is assumed that patients receiving either rivaroxaban or acenocoumarol are switched to aspirin monotherapy.

*SOT* safety on treatment, *QALYs* quality-adjusted-life-years, *LYs* Life-Years, *NA* not applicable, *Riv. Dom* Rivaroxaban is dominant, *ICER* incremental cost effectiveness ratio, *VKA* vitamin-K-antagonist.

### Sensitivity analyses

Sensitivity analysis showed that rivaroxaban was a dominant alternative over VKA when data derived from ITT analysis of ROCKET AF trial was considered in the model (Table 4). Moreover, in the case where patients treated with rivaroxaban and VKA were assumed to be switched to no treatment, rivaroxaban remained a dominant and a cost-effective option over VKA when SOT and ITT data were considered in the model, respectively (Table 4). Additionally, when excluding the VKA-related disutility, the analysis revealed that rivaroxaban remained a dominant option versus VKA either in case of the SOT or the case of the ITT approach.

**Table 4:** Cost-Effectiveness results for sensitivity analyses

**Based on ITT data of ROCKET AF trial (switch to aspirin for rivaroxaban and VKA-treated patients)**		
	**Rivaroxaban**	**VKA**
Total costs (€)	7,927	8,020
QALYs	6.49	6.29
ICER/QALY Gained (€)	NA	Riv. Dom
**Based on SOT data of ROCKET AF trial** (switch to no treatment for all patients)		
Total costs (€)	9,952	10,029
QALYs	6.24	6.04
ICER/QALY Gained (€)	NA	Riv. Dom.
**Based on ITT data of ROCKET AF trial** (switch to no treatment for all patients)		
Total costs (€)	9,597	9,554
QALYs	6.29	6.10
ICER/QALY Gained (€)	NA	233

*SOT* safety on treatment, *ITT* intention to treat, *QALY* quality-adjusted-life-year, *NA* not applicable, *VKA* vitamin-K-antagonist, *Riv. Dom* Rivaroxaban is dominant.

The analysis conducted in specific sub-populations showed that rivaroxaban remained a dominant alternative over VKA in patients at high risk of stroke, those with prior stroke, and those being VKA-naïve.

Additionally, as mentioned earlier, rebates are applied to drugs which are reimbursed and are included in the positive drug list. When the rebate of 9% (entry rebated) or 14% (cumulative rebate based on the entry one and additional 5% based on sales) was incorporated into the calculation of daily drug cost, rivaroxaban was found to be much more cost saving alternative relative to VKA (-€468 and -€650 for 9% and 14% respectively).

Extensive one-way sensitivity analyses were carried out in order to identify the key drivers of cost-effectiveness. With regards to the comparison between rivaroxaban and VKA, the key drivers of cost-effectiveness based on cost/QALY gained were the VKA related monitoring visits during maintenance, the discontinuation and subsequent discontinuation rate with rivaroxaban and acenocoumarol, the VKA-related utility decrement, rivaroxaban effectiveness, the RR for rivaroxaban versus VKA for stroke and MI, VKA monitoring costs at intitiation of therapy and follow up and the transportation cost (Table 5).

**Table 5:** One-way sensitivity analysis for ICER of rivaroxaban vs. VKA (acenocoumarol)

	**Base case value**	**Low value**	**High Value**	**ICER/QALY gained low**	**ICER/QALY gained high**
Acenocoumarol monitoring maintenance visits	3.00	1.00	5.00	€8,635	Riv. Dom.
Subsequent discontinuation rate riva	0.04	0.00	0.09	€7,630	Riv. Dom.
Utility decrement: stable on acenocoumarol therapy	0.95	0.92	1.00	Riv. Dom.	Riv. Dom.
Discontinuation rate riva	0.09	0.00	0.54	€390	Riv. Dom.
Subsequent discontinuation rate acenocoumarol	0.04	0.00	0.06	Riv. Dom.	€942
Discontinuation rate acenocoumarol	0.08	0.00	0.31	Riv. Dom.	€3311
Myocardial infarction RR for rivaroxaban	0.81	0.63	1.06	Riv. Dom.	Riv. Dom.
Rivaroxaban effectiveness	1.00	0.75	1.25	Riv. Dom.	Riv. Dom.
Stroke RR for riva	0.94	0.75	1.17	Riv. Dom.	Riv. Dom.
Acenocoumarol monitoring cost – follow	22.00	17.69	26.31	€228	Riv. Dom.
Acenocoumarol monitoring cost-initiation	32	25.73	38.27	Riv. Dom.	Riv. Dom.
Transport need (% of patients)	50%	0%	100%	€10,388	Riv. Dom.
Other therapy monitoring visits	0.25	0	1	Riv. Dom.	Riv. Dom.

*VKA* vitamin-K-antagonist, *Riv. Dom* Rivaroxaban is dominant, *ICER* incremental cost effectiveness ratio, *QALY* quality-adjusted-life-year, *RR* Relative Risk.

#### Probabilistic sensitivity analysis

**Figure 2:**
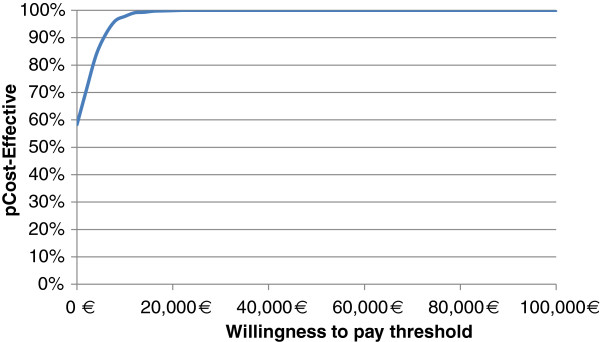
Probability of rivaroxaban being cost effective at alternative willingness to pay thresholds: Acceptability Cost Effectiveness Curve.

The probabilistic sensitivity analysis showed that there is an almost 58% probability of rivaroxaban dominanting VKA. Moreover, the likelihood of rivaroxaban being cost-effective at a WTP threshold of €30,000/QALY was found to be 100% in relation to acenocoumarol (Figure 2).

## Discussion

In the present study, an economic evaluation was undertaken to compare, from a payer perspective, rivaroxaban relative to acenocoumarol for the prevention of stroke in patients with AF in Greece. For this purpose, an existing Markov model was adapted with local data. The input data of the model was extracted from the ROCKET AF trial, other published studies, experts opinion and local government sources.

The analysis showed that rivaroxaban was a less expensive and more effective therapy over acenocoumarol. These results held true in extensive sensitivity and probabilistic analyses. Our findings are in line with those obtained from a recent economic evaluation conducted from a UK NHS perspective, based on the model used in the present analysis. The ICER of rivaroxaban versus warfarin ranged between £2,870 per QALY gained, including warfarin-related disutility and £29,500 per QALY gained, excluding warfarin-related disutility [17]. Moreover, another economic evaluation of rivaroxaban compared to dose-adjusted warfarin for the prevention of stroke in patients with AF from a US payer perspective and a lifetime horizon has been published [23]. This study showed that treatment with rivaroxaban is related to an additional cost of $27,498 per QALY gained over warfarin, indicating that rivaroxaban may be a cost-effective alternative for stroke prevention in AF. Finally, a recently published cost-effectiveness study from a Belgian healthcare payer perspective, showed that the use of rivaroxaban resulted in an incremental cost-effectiveness ratio of €8,809/QALY and 7,493/LY gained [22].

When new treatments are approved for use in health care systems, information is often limited and thus models like the one described here present the only option to evaluate their economic and health outcome impact. Models are also necessary because trials are too simple and short to capture reality in the long run. The analysis was based on an adaptation of an international model, following certain methodological standards reported in the literature. However, it still suffers from several limitations and it should be viewed in that light. It does not represent real world evidence of new oral anti-coagulants, but instead it is based on the synthesis of data reported in a RCT and in the literature that introduce uncertainty in the results. Although the methodology adopted followed the standard recommendations as well as conducting various sensitivity analyses to deal with uncertainty, it cannot substitute for real-life direct comparisons amongst the alternative treatments. Hence, post-launch observational studies are needed to verify the conclusions obtained from analyses such as the present one. A more complete analysis of real-world efficacy and real-world prescribing behavior as well as a broader (societal) analysis may be worthwhile. True health care and patient direct and indirect cost are higher than those used here, and therefore the cost-effectiveness of the new therapy may be more favourable from a societal perspective. Moreover, in the present analysis it was assumed that the clinical outcomes obtained from the ROCKET AF trial were applicable to the Greek health care setting. The use of this data may be questionable, however given the lack of local related data, this choice was the only source of relevant clinical data; one may argue that pivotal trials are almost universally used to build models for pricing and reimbursement decisions. In addition, in the present study, it was assumed that the efficacy and safety of acenocumarol was identical to warfarin in ROCKET AF study and they differ only in terms of their price. Based on a literature search and expert advice, acenocoumarol and warfarin do not appear to have any statistically significant differences in terms of total number of patients within the therapeutic range and in terms of their efficacy and use in clinical practice [42-45]. It should be noted that the results have to be considered in the strict Greek setting and on the basis of the present time resource and drug prices. If any of the underlying parameters change, so may the results and the conclusions of the analysis.

## Conclusion

In conclusion, the present economic analysis suggests that rivaroxaban for AF patients is associated with a greater health benefit and a lower lifetime cost relative to acenocoumarol. Therefore, rivaroxaban may constitute a dominant alternative for prevention of thromboembolic events in AF patients, in Greece and as such policy makers should add in rivaroxaban in the reimbursement list to ensure patients access to this therapy.

## Competing interests

NM received unrestricted grant from Bayer Hellas. However, the study sponsor had no interference in the study design, data collection or writing of the manuscript. None of the rest of authors has any personal or financial competing interest.

## Authors’ contributions

G.K. adapted the model, conducted the analyses, interpreted the results and wrote the manuscript. NM supervised the study, contributed to results interpretation, and he is the guarantor for the overall content. GA and PV were the local clinical experts contributed to data validation and results interpretation. All authors reviewed the manuscript. All authors read and approved the final manuscript.
